# An ethical code for collecting, using and transferring sensitive health data: outcomes of a modified Policy Delphi process in Singapore

**DOI:** 10.1186/s12910-023-00952-7

**Published:** 2023-10-04

**Authors:** Tamra Lysaght, Hui Yun Chan, James Scheibner, Hui Jin Toh, Bernadette Richards

**Affiliations:** 1 Centre for Biomedical Ethics, Clinical Research Centre, Yong Loo Lin School of Medicine, National University of Singapore, Level 2 Block MD11, 10 Medical Drive, Singapore, 117597 Singapore; 2 College of Business, Government & Law, Flinders University, Ring Road, Bedford Park South Australia 5042, GPO Box 2100, Adelaide, South Australia 5001 Australia; 3 Academy for Medical Education, Medical School, The University of Queensland, 288 Herston Rd, Herston, QLD 4006 Australia

**Keywords:** Health data ethics, Bioethics, Ethical guidelines, Health data, Data sensitivity, Modified Policy Delphi, International data transfer

## Abstract

One of the core goals of Digital Health Technologies (DHT) is to transform healthcare services and delivery by shifting primary care from hospitals into the community. However, achieving this goal will rely on the collection, use and storage of large datasets. Some of these datasets will be linked to multiple sources, and may include highly sensitive health information that needs to be transferred across institutional and jurisdictional boundaries. The growth of DHT has outpaced the establishment of clear legal pathways to facilitate the collection, use and transfer of potentially sensitive health data. Our study aimed to address this gap with an ethical code to guide researchers developing DHT with international collaborative partners in Singapore. We generated this code using a modified Policy Delphi process designed to engage stakeholders in the deliberation of health data ethics and governance. This paper reports the outcomes of this process along with the key components of the code and identifies areas for future research.

**Supplementary Information:**

The online version contains supplementary material available at 10.1186/s12910-023-00952-7.

## Background

Digital Health Technologies (DHT) encompass a wide range of data-driven tools and applications spanning from wearable devices to clinical decision support software [1]. DHTs also play a key role in gathering large amounts of clinical, lifestyle and behavioural data to drive new strategies in healthcare, such as precision medicine, and transform the delivery of health services by reducing pressure on scarce hospital services and into self-managed care [2]. To achieve these goals, DHT will rely on researchers collecting, storing and curating large datasets linked from multiple sources and transferring them to health professionals and researchers at different institutions and potentially, across jurisdictional borders. A key concern to some is the highly personal and potentially sensitive information about the health of individuals contained within these datasets [3]. This study addresses some of the ethical and legal challenges in the sharing and transfer of such datasets.

Researchers developing DHT in cross-jurisdictional collaborations should not only comply with relevant laws and institutional requirements within their jurisdiction but also with those applicable to their international collaborators. In addition, they must meet ethical standards (and community expectations) for the collection, use and transfer of potentially sensitive health data applicable to the specific cultural frame within which this sharing takes place. Care must be taken to avoid assumptions of what is acceptable based upon local knowledge only, as acceptable standards are context-dependant and vary according to local norms, beliefs and values. This study aims to inform and support the sharing of data within the Singaporean context and provide an exemplar of culturally appropriate use of data to inform DHTs.

Complying with legal requirements and meeting ethical standards is critically important for two reasons. First, legal liabilities incurred through the unauthorised disclosure of confidential information and subsequent breach of privacy may incur financial penalties as well as a loss of public trust in the institution(s) responsible for the data. With a decline in public trust comes a reticence to share data and a dilution of breadth of key datasets. These incursions serve to undermine the efficacy of the DHTs and, in turn, lead to a failure to meet any identified goals. A further complexity arises if (or when) the process of data collection and usage falls short of accepted moral expectations, which can incur an ethical debt and undermine the social license [4].

The social licence refers to practices that communities are willing to accept as morally and socially permissible [5], and an ethical debt arises when researchers fail to identify and respond appropriately to the expectations of publics and other stakeholders [6]. An ethical debt has been likened to technical debt, which “occurs when an organization opts for an easy, sub-optimal software solution in order to economise resources [7]and time in the near term, with a vague notion of spending time in the future to fix it.” Ethical debt thus arises when the social licence is breached due to haste or lack of care in the use and development of technology. It cannot, however, simply be repaid and is evidenced by a loss of trust, which can be difficult to rebuild and may undermine the feasibility of research initiatives.

The collapse of the UK care.data in 2013 is an example of how ethical debt can be incurred and lead to a loss of social license and demonstrates the ongoing harms that can arise when public expectations are not met. In this example, the UK government passed legislation that created an ‘Information Centre’ which was “a body corporate with the power to collect, collate and provide access to the medical information for *all patients* treated by the NHS in England.” [8] This extended the already accepted collection of data by hospitals and GP Clinics to the collection of this data for access by unknown parties for unarticulated use. Overall, there was a lack of clarity around the protection of the data and what would be the parameters around access.

The response from the public was swift and strong and the scheme was quickly ‘paused’ with plans to reintroduce it within a few months, yet the public resistance proved too strong and care.data was ultimately put aside. The 2021 General Practice Data for Planning and Research proposal was an attempt to revive the scheme adopting the established tag line ‘data saves lives’. However, this proposal faced similar challenges and was paused before the planned program launch in 2021. Of significant concern was the lack of transparency along with potential commercialisation of personal data, despite broad recognition of the potential benefits to be accrued from the program [8].

The overall public resistance to these proposals came about despite general recognition of potential for good through the sharing of data. However, the harm done (ethical debt incurred) by the lack of transparency when care.data was originally introduced means there is an ongoing lack of trust and social license for data sharing schemes in the UK. The key takeaway is that if the transformative goals of DHT are to be achieved, the mistakes of care.data must be avoided. Thus, it is imperative for researchers to understand public expectations and align their practice with accepted moral and ethical norms when collecting, using, and transferring potentially sensitive health data. This study addresses this and facilitates an informed approach to DHT through the generation of ethical guidance for researchers collecting, using and transferring potentially sensitive health data in Singapore.

### Ethical guidance for health data in Singapore

The Southeast Asian city-state of Singapore is a small, high-income country with advanced digital health infrastructure and well-funded research sectors. To serve its national interests, Singapore relies heavily on drawing scientific expertise into the country and collaborating with strategically important research partners abroad [9, 10]. The collection, use and transfer of health data out of Singapore to collaborative research partners must comply with local personal data protection laws. To avoid ethical debt and compromising social license, these activities should also align with culturally relevant norms and expectations for the conduct of health data research in Singapore. A further layer of complexity is added when cross border transfer is planned as there must be compliance with the laws and cultural norms of the receiving jurisdiction.

In Singapore, the Personal Data Protection Act (PDPA) 2012 protects personally identifiable information and the Human Biomedical Research Act (2015) regulates research on human subjects that collects and stores identifiable health data. However, both laws only apply to personally identifiable information and not to anonymised datasets that may nevertheless contain potentially sensitive information about research cohorts. The PDPA identifies the unauthorised disclosure of medical information as reportable (Sect. 26B). Further, Part 1 of the Schedule to the Personal Data Protection (Notification of Data Breaches) Regulations 2021 sets out a list of personal data and circumstances in which breaches are reportable to the affected individuals and the Personal Data Protection Commission (PDPC). The prescribed personal data relates to personally identifiable information, such as financial details and health status of individuals. The Ministry of Health (MOH) [11] has clarified the types of medical information that are reportable under the PDPA (such as those stored in electronic medical records). However, this guidance only applies when the medical information disclosed is personally identifiable.

In the absence of clear legal and regulatory pathways to mitigate risks of ethical debt, our research aimed to generate an ethical code to guide the collection, use and transfer of potentially sensitive health data for DHT research in Singapore. Many guidance documents and ethical frameworks for health data governance have been published internationally [12–17]. Notable examples include the *Framework for Responsible Sharing of Genomic and Health-related Data* developed by the Global Alliance for Genomic and Health aimed at the governance of genomic and related data, *A Code of Digital Ethics* [18], the *Systemic Oversight Model* for health data [19, 20] and the *Health Data Governance Principles* developed by a collective of global organisations and contributors.

International bodies, such as the UNDG and OECD, that have developed guidelines to address personal data protection and security issues are underpinned by broad common principles such as privacy, lawful and fair use, transparency and accountability [21, 22]. These frameworks are often broadly devised and remain abstract for implementers and researchers to operationalise in their decision-making. Although Becker’s *Code of Digital Ethics* refers to data generated across a range of business products and technological solutions intended for implementation in business, it does not provide specific guidance for health data. In other words, these documents are not directed specifically at guiding research with certain sources of health information that may (or may not) be considered potentially sensitive in any particular context.

**Fig. 1:**
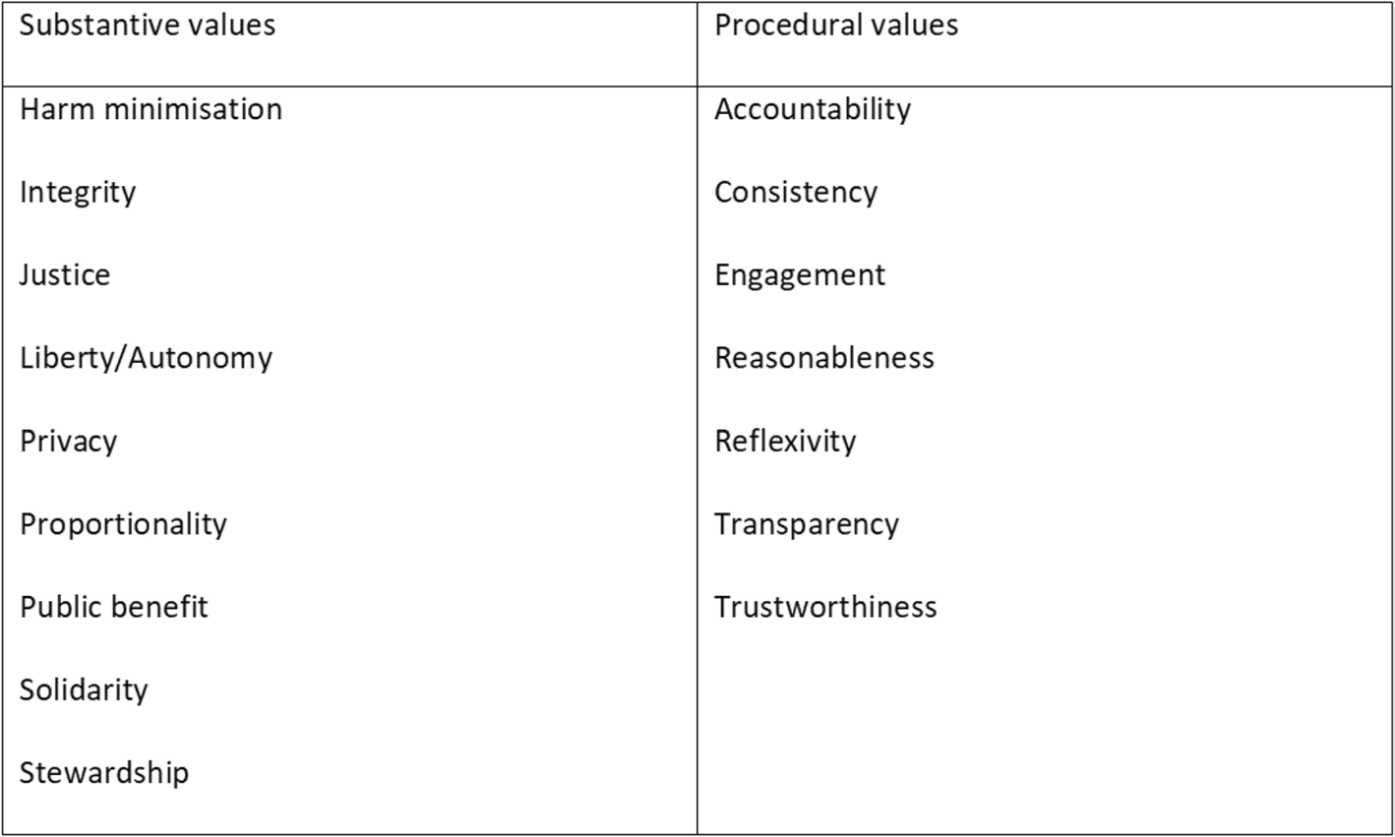
Ethical values in the Big Data Ethics Framework

Additionally, few guidance documents are specific to Southeast Asian contexts. This gap is important to address because while ethical principles may apply generally, the weight or priority attached to their underlying values can differ across contexts. The *Ethics Framework for Big Data in Health and Research* [23] is one notable exception. This framework identifies sixteen substantive and procedural values (shown in Fig. 1) that can be drawn upon in addressing the ethics of specific use cases. It was developed with an international group of experts under the auspices of the *Science, Health and Policy-relevant Ethics, Singapore* (SHAPES). Not all values will apply to every situation. However, the framework is useful a priori starting point for generating an ethical code that is both values-based and empirically informed on how researchers should collect, use and transfer whatever constitutes potentially sensitive health data in this context of Southeast Asia.

To generate the code, we engaged with stakeholders and researchers at the Future Health Technologies programme. This collaborative research partnership aims to develop scalable DHT for use in Singapore with partner investigators in both countries [24]. The research programme is broadly governed under the systemic oversight model [20], which promotes principles of AFIRRM (adaptivity, flexibility, inclusiveness, reflexivity, responsiveness and monitoring) in the collection, use, linkage, storage and sharing of health data at an operational level. Adopting an approach outlined in Becker et al. [18], our study drew on AFIRRM’s broad governing principles to codify the SHAPES values-based framework into ethical guidance with stakeholders in a modified Policy Delphi process.

## Methods—Modified Policy Delphi process

Our study adapted a modified Policy Delphi process designed to deliberate with stakeholders on the development of data governance frameworks [25]. The Delphi method is a process of systematically building consensus with a panel of experts and/or stakeholders over successive rounds of prioritisation and voting. The Policy Delphi is useful for establishing policies and guidelines in areas with limited evidence and where expert opinion is crucial [26]. The modified version of the Policy Delphi does not seek to achieve consensus but leaves open the possibility for multiple options and dissention amongst panellists [27]. Following the Majumder et al. (2021) [25] design, our modified Policy Delphi engaged with an expert panel of researchers and other stakeholders over three stages of mixed methods research starting with: 1) semi-structured interviews with panellists to generate statements and policy options, 2) an online survey for panellists to prioritise statements and options generated from the interviews, and 3) a deliberative workshop with the panel to deliberate on the definition of sensitive data and generate an ethical code.

The mixed methods design combines qualitative and deliberative methodologies that are not intended to produce generalizable findings from large representative populations but to generate an in-depth and rich account of perspectives, beliefs and experiences about social phenomena [28, 29] or, in this setting, ethical norms and standards. Correspondingly, sampling strategies are aimed at recruiting participants that are broadly inclusive of target populations, rather than being representative, and in small numbers to facilitate dialogue and deliberation on complex normative questions [27]. Our Delphi panel was recruited using purposive and snowballing sampling methods through the research networks of the investigators and the research programme according to one of the five stakeholder groups identified in Majumder et al. (2021) [25]: (1) data contributors (i.e. patient advocates); (2) data generators (i.e. programme researchers); (3) data facilitators (i.e. regulators and data security managers); (4) data resources (i.e. data custodians and access controllers); and (5) professional data users (i.e. clinician and industry partners). Panellists were asked to commit to all three stages of the process and were compensated with tokens of appreciation for the contributions. The process was reviewed and approved by the National University of Singapore – Institutional Review Board.

### Stage 1: Semi-structured interviews

The semi-structured interviews were conducted from April to July 2022 with 28 panellists; 13 were completed in-person and the remaining virtually. The interviews explored ideas about health data sensitivity, acceptable data linkages and international data transfer, as well as potential policy options (see interview guide in Supplementary Material 1). Panellists were sent information about health data ethics and governance in Singapore two weeks in advance of the interview [30]. Interviews were recorded and transcribed for qualitative content analysis in NVivo Software [31], using both deductive and inductive methods. The research team inductively identified statements from the interviews about data sensitivity and sources of health data that panellists considered to be potentially sensitive. These sources were contrasted with and added to the MOH list of reportable medical information under the PDPA. For the deductive analysis, we applied the SHAPES framework (Fig. 1) to extract statements to enact the values in the ethical code.

### Stage 2: Survey

From our analysis of the interviews, we designed an online survey on the Qualtrics (USA) platform. The survey was divided into two sections: (1) defining sensitive health data and, (2) generating statements for the ethical code. For the first section, panellists were presented with the health data sources and sensitivity statements identified from the inductive analysis. On a six-point Likert scale, they rated both the sensitivity of data sources and their level of agreement with each of the statements on data sensitivity. For the second section, panellists were presented with 3–5 statements under each of the sixteen values in Fig. 1, which were defined directly from Xafis et al. [23] and asked to rate (on a Likert scale of four) their desirability and feasibility for the ethical code. Open response options also allowed panellists to add suggestions and comments on the nominal items. See Supplementary Materials 2 for the survey questions.

The survey was completed in September 2022. The data were analysed qualitatively to generate a working definition of sensitive health data and descriptively to prioritise statements for the ethical code that met a threshold of 2/3^rd^ majority (66%).

### Stage 3: Workshop

Results of the survey were presented to panellists at the stakeholder workshop. The workshop was convened in October 2022 as a hybrid event at the CREATE campus in Singapore under the Chatham House Rule. The program was structured in two parts with a combination of plenary and breakout sessions (refer to Supplementary Materials 3 for the schedule). The research team assigned panellists to evenly distribute stakeholders across four tables in the breakout groups (including one hybrid) to deliberate on what constitutes sensitive health data in Session 1, and the value statements for the ethical code in Session 2. The lead investigator and a professional facilitator with local events experience co-facilitated the workshop. Three scholars with international expertise in health data law and ethics were also present to answer questions and help facilitate the breakout groups. Plenary discussions were audio recorded for transcription and the draft report was circulated to panellists after the workshop for final comments and feedback.

## Results—Outcomes of the Delphi process

The composition of the panel according to stakeholder group is shown in Table 1. There were at least four panellists from each group with the highest percentage (29%) being data generators, which represented researchers to whom the ethical code is targeted. All 28 panellists were interviewed and completed the survey. Twenty panellists participated in the workshop although two were only available for half the day (one in the morning and one in the afternoon session), meaning that only 19 panellists were present at any given time: 17 panellists participated in person and the other 3 joined virtually. Panellists voted and agreed upon 70% as the minimum threshold for consensus (i.e. 13 out of 19 votes were needed to reach agreement). Points of disagreement were discussed and, if unresolved, noted for the minority report.

**Table 1:** Composition of the stakeholders

Stakeholder group	Interview (*N* = 28)	Survey (*N* = 28)	Workshop (*N* = 20)
Data contributor	5 (18%)	5 (18%)	1 (5%)
Data generator	8 (29%)	8 (29%)	8 (40%)
Data facilitator	6 (21%)	6 (21%)	5 (25%)
Data resources	4 (14%)	4 (14%)	4 (20%)
Professional data user	5 (18%)	5 (18%)	2 (10%)

### Health data sensitivity

At the beginning of the workshop, the panel were shown responses to the survey items on data sensitivity and the various datapoints were discussed alongside data security measures, such as de-identification. Following deliberations, the panel agreed on the following definition for sensitive health data to mean:

*“The health data, of an individual or in aggregate, that exposes persons, groups or populations to an increased risk of harm (e.g., reputational, discriminatory, financial). The risk of harm may be mitigated when, for example, personal identifiers are removed.”*

**Fig. 2:**
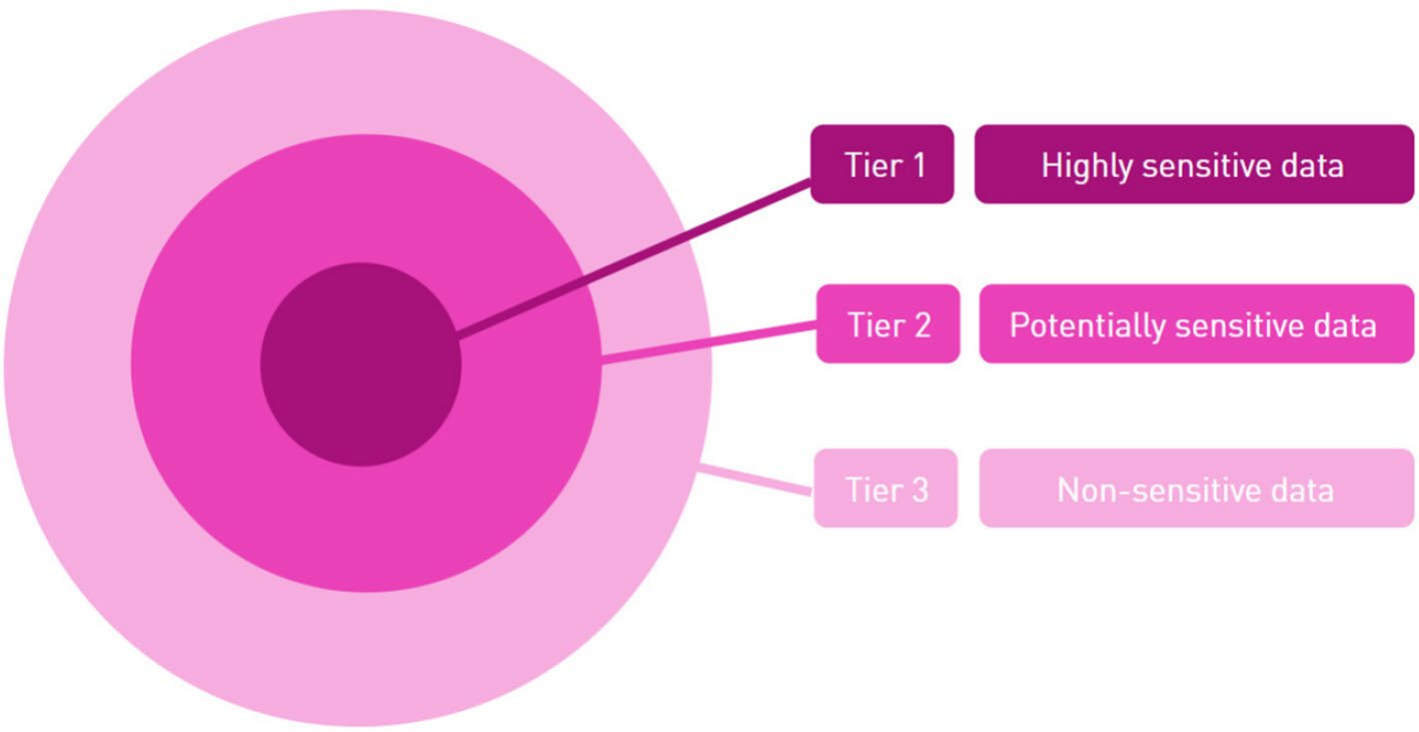
Tiered list of health data points in terms of sensitivity. Refer to https://fht.ethz.ch/research/health-data-governance-value-creation.html for the detailed diagram

The panel also agreed on the tiered list of health data points shown as Fig. 2: highly sensitive, potentially sensitive, and non-sensitive. Tier 1 has the highest degree of sensitivity and includes *personal data* about stigmatising conditions such as, HIV status, history of sexually transmitted diseases (STDs), mental health disorders, and fertility treatments. This ranking generally corresponded with the MOH list of reportable information, except for genome sequencing data and genetic test results, which were not listed at the time. The panel categorised the non-clinical information on the MOH list (i.e. history of suicide attempts and domestic/child/sexual abuse) as highly sensitive. The panel also added medications that could imply sensitive information about a person’s health status (e.g. prescriptions for HIV, STDs, mental health disorders etc.).

The panel agreed that Tier 1 health data would become less sensitive when appropriate security protocols for de-identification or pseudonymisation were introduced. These data would then be considered as potentially sensitive (Tier 2), along with other less sensitive information that has not been deidentified. Tier 2 data included other information on the MOH list, such as drug and alcohol abuse, history of organ transplantation and contraceptive treatments. The panel added medical history of cancer and other common (non-stigmatising) diseases, medical images and self-reported mental health status. Other potentially sensitive data included sexual orientation, voice recordings and other biometric data that DHT researchers might collect. All other non-sensitive data and de-identified Tier 2 data (with possible exception of identifiable biometric data) was categorised as Tier 3 (e.g.: medical history of common diseases (e.g. diabetes, stroke, heart attack etc.), medical images or scans of body parts, a person’s direction-finding ability collected from phone apps).

While the panel was in general agreement with these categories, some areas of disagreement are notable. Although the panel voted to include genetic information in Tier 1, it was not unanimous with one panellist voting against this decision and two abstaining. The dissenting panellist reasoned that this information should be considered no more or less sensitive than any other medical information that may be routinely found in electronic health records.

Although the panel agreed to categorise biometrics (voice/speech, geo-location) as Tier 2, there was disagreement about the relevance of these datapoints to health or whether de-identification could reduce its sensitivity. There was also disagreement about the relevance and sensitivity of adoption status. One panellist suggested that this information has cultural significance within Asian families and should be treated as highly sensitive. Other datapoints were suggested (e.g. surgical procedures, communicable diseases, autoimmune diseases, history of COVID-19, medical allergies, etc.) but were too broad and needed more refinement than time permitted to categorise with any consensus.

Although what counts as sensitive data is a subjective consideration, data sensitivity has become increasingly less straightforward to classify in a big data environment with data linkage. The interconnected nature of data and the immense volume involved pose challenges in determining the level of sensitivity. When data is combined from multiple sources, the resulting dataset can contain information that was not sensitive on its own but becomes sensitive when combined with other data.

### Ethical code for the collection, use and transfer of health data

In the second part of the workshop, the panel broke into smaller groups of four. Each group was assigned with four values from the SHAPES framework [23] and given written materials showing survey responses, including qualitative comments. They were asked to refine and propose one preferred statement for each value and given an instruction that it should be practically feasible to implement in research practice. That is, the statements should not set unattainably high standards that are impossible for researchers to meet in practice. They were also given the freedom to rename, add or remove any of the values, provided they could justify their reasoning to the panel when they reconvened for the plenary discussion. The panel voted on each proposed statement and any residual statements were placed into a more detailed description of the value.

**Fig. 3:**
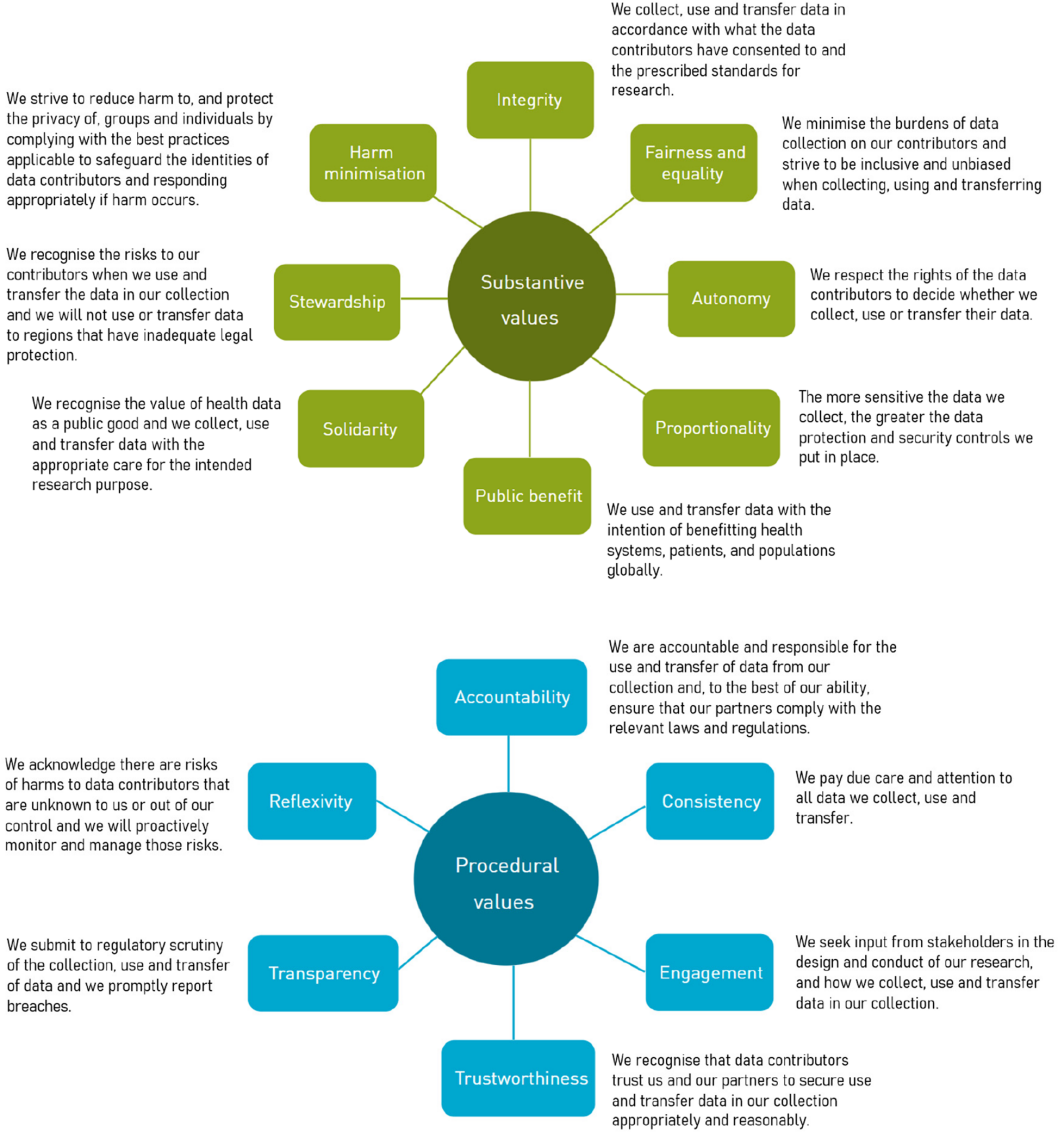
Values and value statements from the SHAPES framework that were voted by the stakeholders as applicable in the collection, transfer and use of data in DHT. See Appendix 1 for the detailed description of the value statements and https://fht.ethz.ch/research/health-data-governance-value-creation.html for the detailed diagram

Following refinement and deliberation, the panel voted on statements for 14 of the 16 values from the SHAPES framework, which are shown in Fig. 3 along with more detailed descriptors for each value. The panel decided to remove two values (privacy and reasonableness) and rename one of them (justice). Justice was renamed after discussion about the complexity of the concept both as a value for restoration or remedy, as well as promoting fairness and equality. While the need for restoration of harms that may occur was acknowledged, it was distinctive from the ideas of inclusion and participation in research that the panel has also proposed. Thus, the restorative notions of justice were folded into the value of harm minimisation and justice was renamed ‘Fairness and Equality’ to encapsulate the latter ideas more clearly.

Reasonableness was removed because the proposed statements and subsequent discussion were not regarded as substantively distinct from the other values. Instead, the concept was folded into the value of trustworthiness. That is, researchers could be expected to take reasonable steps to ensure data is secured and used appropriately, and that their research partners are trustworthy, but the provision of absolute guarantees would be unfeasible to implement. Privacy was removed because the panel reasoned the value was adequately captured within the statements for autonomy and harm minimisation. The statement for harm minimisation explicitly refers to protecting the privacy of individuals and groups through data security measures while the statement for autonomy implies respect for individual legal and moral rights to control access to information about them. Autonomy also refers to respecting those rights by obtaining consent and using data within scope of the consent given to researchers. Hence, the panel did not propose setting a separate value for informed consent.

The values in the ethics framework were developed collaboratively by an international working group convened by the Science, Health and Policy-relevant Ethics in Singapore (SHAPES) initiative. The framework has benefitted from expert input from Singapore and around the world. The values in the framework were presented to the participants in the surveys for their ranking and prioritisation. In the survey, in addition to providing feedback to each of the values, participants had the option to propose other values that they consider important or appropriate for the study for deliberation in the final stage (workshop). During the workshop, participants deliberated and voted for values that they consider feasible and desirable for health research, with the option to rename, add or remove any values presented to them.

## Discussion

Our study aimed to generate an ethical code to guide the collection, use and transfer of potentially sensitive health data for research. Research with personally identifiable data is a social good as it could lead to positive outcomes and advancements for society at large across various fields. Some potential positive outcomes in healthcare include drug discovery and development, precision medicine (i.e. offering tailored treatment to increase the effectiveness of therapies and minimise adverse side effects), and development of evidence-based social policies that leads to better-targeted health interventions and services for vulnerable populations and marginalized communities.

Although several principled frameworks for DHT research already exist, our modified Policy Delphi process adds legitimacy to the abstraction of ethical norms and values into concrete statements to guide researchers. We generated our code in an iterative multi-stage process of stakeholder engagement and deliberation to account for the socio-cultural nuances relevant to the health data ecosystem in Singapore. This process allowed stakeholder groups who have an interest in health data governance to generate an agreed definition of sensitive health data and contribute to setting the ethical standards that researchers in Singapore can reasonably be expected to meet when collecting and using these datasets.

While ethical guidance is often construed as being aspirational in orientation [32], it was important that the value statements in our code were not only desirable [33], but also feasible to implement in practice. In particular, researchers and research institutions should ideally guarantee the protection of data contributors and their identities. However, there will always be a potential for data breaches with or without data sharing, as evidenced by large scale data breaches in Singapore [34, 35]) and abroad [36]. Accordingly, the code for harm minimisation was limited to compliance with current best practices and responding appropriately if and when harms do occur. Similarly, while researchers can be expected to conduct scientifically and socially valuable research, they cannot ensure the outcomes of their research translates into actual public benefits. Hence, the value of public benefit was codified to guide researchers to use and transfer data with the *intention* of benefitting health systems, patients, and populations, rather than promising anything tangible as such an expectation would be unrealistic.

### Socio-cultural nuances

The deliberations revealed interesting socio-cultural nuances with the de-emphasis on privacy as a primary value. Although much of the international literature emphasises privacy as the central ethical concern with health data research [37–39], research in Asia [40], and Singapore specifically, does not correspond with this focus. While Singaporeans do value and guard their privacy, previous studies suggest Singaporeans are more concerns of justice with matters of fairness and ensuring the benefits and burdens of health data research are distributed fairly, and that it is governed with transparency and accountability [33, 41, 42].

Moreover, government agencies are excluded from the PDPA and the population is routinely surveilled for public health, infrastructure management, law enforcement and national security purposes [43–45]. Citizens in many European and North America countries would not likely accept the degree of surveillance Singaporeans have become used to, and largely accept in exchange for social and economic stability and population-wide access to public goods, such as housing, education and security [46]. Thus, the decision to remove privacy from the codified values and encapsulate it instrumentally within harm minimisation (i.e. the subject of protection and restoration rather than an intrinsic right), is consistent with local norms and expectations.

Relatedly, informed consent is prominent in the international literature and in many ethics frameworks [47–49], but was not emphasised in our code. This outcome partly resulted from our starting point with the SHAPES ethics framework, which did not explicitly include informed consent as a specific value because of its diminishing and increasingly impractical role in big data research [23]. Instead, the SHAPES framework placed primacy on *respect for persons* as the more important value that underpins many others, including privacy and informed consent. Panellists in our study similarly did not prioritise consent but embedded it in the relevant values of autonomy and integrity. This stance is consistent with prior empirical studies in Singapore [41, 42] suggesting that while an opt-in consent would be necessary, it is insufficient and other, more important values, are needed for the trustworthy collection, use and transfer of health data for research.

Another nuance from our process was the explication of justice as fairness and equality to distinguish the value from legal concepts of restorative or retributive justice. This emphasis aligns with the Rawlsian conceptualisation of justice [50, 51] as fairness and the equitable distribution of benefits and burdens from research. It also corresponds with prior empirical studies in Singapore where equity, inequality and fairness have been reported as persistent concerns with health data sharing [41, 42]. The restorative elements of justice in our code were folded into the value of harm minimisation in recognition of the need to respond appropriately and remedy harms caused from the collection, use and transfer of sensitive health data.

Finally, our process revealed important nuances and potential gaps in the regulation of sensitive health data in Singapore. Most strikingly was a lack of an explicit reference to genetic test results and genome sequencing data from the list of medical information where, if breached, must be reported under the PDPA. Internationally, genetic information is generally understood as sensitive [52] and, in some European jurisdictions (such as Switzerland) receives exceptional protection under data protection laws [53–57]. The sensitivity of genetic information relates to its potential to reveal personal and familial information about someone’s health status, ethnicity, and risks to certain illnesses, especially when linked to other datasets [52].

Not all genetic information is seen as particularly sensitive (such as somatic cancer gene test results) and some believe it should be treated like any other clinical data that may be even more sensitive, such as mental health status and history of substance abuse [52]. However, others regard whole genome sequencing data as being particularly sensitive given the depth of information it can reveal about an individual and their relatives, and the increasing challenges in truly de-identifying these datasets [58, 59]. Although Sect. 26B(2) of the PDPA suggests that the list of prescribed sensitive health information is not exhaustive, it is unusual that genetic data, given its aforementioned sensitivity, is not explicitly included.

The sensitivity of several other data points was raised throughout our study but no agreement was reached. The relevance and sensitivity of adoption status was particularly contentious. Some studies [60–63] have revealed societal stigma regarding adoption, although these attitudes have changed to become less stigmatising over time. Likewise, research exploring the experiences of adults conceived by sperm donation [64, 65] revealed challenges with addressing stigma regarding male infertility and difficulties in disclosing the child’s donor.

Other data points were suggested (e.g. surgical procedures, communicable diseases, autoimmune diseases, history of COVID-19, medical allergies, etc.) but were too broad and would need more refinement to categorise with any consensus. The panel deliberations over these points of disagreement highlighted the importance of context in the collection and use of health data. This implies that health data on its own may not necessarily be sensitive, however, the context in which they are used may render them sensitive. For example, whilst Covid-19 infection status was potentially stigmatising in the early stages of the pandemic, this status became far less stigmatising as the disease became endemic in the population. Similarly, infectious diseases such as TB, hepatitis or HIV (including medication to treat HIV) that may cause stigmatisation are considered sensitive in the society in which these operate as they could cause social exclusion to the affected individual. The concern for re-identification remains important, where the risks of re-identification is high due to data mismanagement, less sensitive data may become sensitive when linked with other datasets.

### Regulatory and legal implications

Our study demonstrated the need for clearer legal and regulatory pathways for the collection, use and transfer of potentially sensitive health data that are de-identified but could be re-identified in combination with other available datasets. Existing guidance on data sensitivity classification issued by the Singapore MOH lists a range of specific information (e.g., history of sexually transmitted diseases, mental health status and reproductive health interventions such as pregnancy termination and fertility treatments) as sensitive but does not address the concerns raised by our study panellists as outlined above. Although the PDPA 2012 governs the collection and use of personal data, including provisions for transfer limitation obligations, the sensitivity of personal data is not directly connected to whether it can be transferred between data controllers or across jurisdictional boundaries. This gap between the law and ethics represents an area where researchers are at risk of incurring an ethical debt, and compromising the social license, when participating in cross border research activities involving potentially sensitive health data.

Pathways for the legal transfer of data from Singapore include the consent of patients, contractual agreements or binding corporate rules. The latter two pathways need to guarantee that the data will receive equivalent protection under Singaporean law. These requirements exist irrespective of the sensitivity of the data, which will determine the appropriate security measures required for different types of data.

### Multiple tiers of health data sensitivity

There is a distinct scale as to what counts as sensitive, with classification of diseases less sensitive compared to patient’s genetic information and physiological data [66]. Additionally, information such as genomic data, which at present is not considered as distinctly sensitive, may be potentially sensitive in the future. Data sensitivity could be further classified according to socio-cultural perceptions; disease or illness; purpose of use or access; religious or familial perspectives; intrinsic worth; commercial viability, or what the law designates as sensitive [52, 67–70].

From the governance perspective, the EU General Data Protection Regulation (GDPR) does not specifically define what constitutes sensitive health data. However, it defines “sensitive” personal data as personal data related to race or ethnicity, political opinions, and religion; trade-union membership; genetic and biometric data; health-related data; and data that identifies sexual orientation (Article 4). The GDPR then prohibits processing such sensitive personal data unless specific measures are taken in certain circumstances. These circumstances include where the data subject has given consent, or where the data is being used for research, statistical or public health processes (Articles, 9(2)(a), (2)(i), 2(j); recitals 51–54 GDPR).

In Singapore, the PDPA does not explicitly define sensitive data, but PDPC guidelines stipulate that security controls should be adopted for personal data commensurate to the level of sensitivity. Similarly, these guidelines seem to leave open the possibility of including other forms of personal data unto the category of sensitive health data. This includes sensitive health information generated through inference with the use of derived data, user activity data or user provided data (Advisory Guidelines on the Personal Data Protection Act for Selected Topics, 2022).

Empirical work done in Europe, the United States and Australia, has noted that sensitive data can extend to a wide variety of information including, but not limited to, varied health-related, demographic and financial information [71–79]. However, some studies emphasised that the definition of data sensitivity should vary with the socio-cultural and economic context of the data donors, in so far as genetic data is concerned [66]. A normative account of what constitutes sensitive health data, including from a socio-cultural perspective is highly relevant in ethnically diverse countries where data sharing programmes continue to develop. Additionally, the expansion of data sharing and data linkages have consequential impacts upon the meaning of data sensitivity, such that despite the best effort in de-identification in the research process, potentially sensitive data may still become re-identified or linked through information already available online.

The lack of clear identification of what constitutes sensitive health information remains challenging to researchers, data controllers and users, amongst others who are engaging in research or governance activities in a big health data ecosystem. Considerations of the meaning of sensitive health data is thus essential in constructing a trustworthy data governance framework. Based on our Panel deliberations and international trends towards the use of tiered systems for data access [80], we suggest the following minimal ethical standards apply to DHT research with potentially sensitive health data:

#### Tier 1—Informed consent

Tier 1 represents highly sensitive personally identifiable health data requiring the highest level of safeguard in terms of security and management, where the explicit informed consent from data contributors must be obtained prior to the collection, use and transfer of such data for research purposes.

#### Tier 2 – broad consent with IRB approval

Tier 2 represents data that are de-identified but could be potentially sensitive when re-identified or linked with other datasets. Tier 2-type health data can be used for research purposes however the broad consent of data contributors should be obtained, supported by appropriate approval from ethics committees or institutional review boards (IRB). This approach aims to strike a balance between the obligations of researchers in seeking consent that is not too onerous to fulfil while keeping contributors informed about the use and transfer of their data.

#### Tier 3 – IRB approved waiver of consent

Tier 3 data contains aggregated de-identified data that are non-sensitive for research purposes where its collection, use and transfer require approval from IRB where such consent could be waived where local laws permit. For Singapore, this waiver would need to be sought pursuant to the *Human Biomedical Research Act*, as well as any other human ethics legislation that applies to research teams in other jurisdictions.

### Limitations and future opportunities

Our study has demonstrated how the modified Policy Delphi process can inform the generation of an ethical code that is normatively grounded and culturally appropriate for DHT research. The small sample size in our process was conducive to the mixed methods approach we adopted for fostering dialogue and seeking consensus. Our findings are not intended to be generalizable beyond the context of DHT research in Singapore although the values we articulated in the ethical code may apply elsewhere given the consistency with other empirical studies and normative frameworks.

Future studies applying this methodology should note the limitations we encountered with the attrition of panellists from Stages 2 to 3. While Stages 1 and 2 had full participation (*n* = 28), nearly 29% dropped out in stage 3 (*n* = 20). Within the patient network/support (data contributors) group, only one participant attended the workshop (stage 3). Although we did not follow up with panellists on their reasons for not attending, the workshop being held on a weekday may have conflicted with work or other personal commitments, unlike the other professional stakeholders. In any case, the regrettable loss of this stakeholder group may affect the desirable inclusion of this data contributors in deliberations and efforts should be made to accommodate their participation as much as possible.

Another question for future studies is how any outcomes might be feasibly implemented. One of the challenges of implementing our ethical code will be determining when different types of data fall into one of the three tiers proposed above. This process will require a continual assessment of the data, particularly with respect to the types of inferences that could be drawn. We recognise that this difficulty is a persistent challenge in research involving big data due to possibilities of linking data from multiple sources, resulting in data that are not normally considered as sensitive to become sensitive. The requirement for continual assessment of data can be considered as a safety net for stakeholders in health research in being accountable to data contributors as best as they could while allowing research to be conducted. Users could regularly review and update the ethical code based on changing best practices, technological advancements, and input from stakeholders. Regular training is also essential for employees and partners involved in data transfer to ensure they understand and adhere to ethical principles, and to ensure compliance with relevant data protection laws. Additionally, the legal challenges associated with cross border data sharing are not directly connected to the sensitivity of personal data. Sensitive health data is subject to the same data transfer requirements as other personal data. Furthermore, there are challenges with determining when data has been anonymised or de-identified and laws in different countries may have different standards for these measures.

According to guidelines published by the PDPC, a dataset will be deidentified when all direct identifiers from that dataset are removed. However, that dataset will still be treated as containing personal data. By contrast, anonymisation involves removing or masking all direct and indirect identifiers from a dataset so there is no serious possibility that individuals can be reidentified from that dataset. Although sensitivity may be relevant for determining the possibility of an individual being reidentified, transfer requirements under Singaporean law apply to all personal data. Other jurisdictions also impose a higher threshold test for determining when data is anonymised. Therefore, a separate legal framework accounting for jurisdictional differences is required to complement our ethical code. This separate legal framework would be consistent with the principle of accountability, and would be an additional research project for our team.

Prior to implementing our code, future studies should also aim to test the relevance and perceived sensitivity of data in each of the tiers we have to ensure the proposed governance measures will meet the expectations of both participants in DHT research and the wider community. Engaging directly with these stakeholders will be crucial for not only applying the ethical code in practice but for securing the social license to collect, use and transfer potentially sensitive health data in this context. Additionally, there were other questions raised during the deliberations but were ‘parked’ due to time constraints. Whilst we managed to conclude on key questions, the opportunity for a deeper discussion on questions raised during the discussion would be valuable to provide further insight into the code of guidance.

Several studies have discussed challenges of operationalising high-level principle-based ethical codes. Examples were (i) new health technologies that emerge faster than the updating of ethical codes [81]; (ii) seen as foreign and inconsistent with cultural and societal context [82]; (iii) lack of sufficient detail (i.e. ambiguities, vagueness and contradictions) in the application to specific ethical dilemmas [82]; and (iv) lack of motivation or enforcement [83]. To address these challenges and apply ethical codes in practice more effectively, suggested approaches in existing literature include: establishing and maintaining ethics committees to provide guidance and support over competing principles [82]; considering all the multidisciplinary stakeholders’ needs during implementation [84]; ethics training program specifically designed to give individuals the opportunity to practice interpreting and applying professional principles of their respective field [83, 85]; better communication of guidelines by developing clearly worded principles and norms [85, 86].

## Conclusion

The use of large datasets for DHT for health research often involve local or overseas transfer for further analysis and processing, sometimes occurring in several jurisdictions. Such activities often raise important ethical and legal considerations necessitating compliance by researchers to avoid incurring ethical debt and legal liabilities. Our study generated an ethical code that articulates substantive and procedural values to guide researchers in the collection, use and transfer of potentially sensitive health data. This code also makes explicit health data points that may be considered highly or potentially sensitive within the context of Singapore and researchers ought to take due care when collecting, using and transferring these data within international collaborative arrangements. Failure to do so may incur an ethical debt and undermine the feasibility of the research.

### Supplementary Information

**Additional file 1: Supplementary Material 1.** Stakeholder Engagement for Trustworthy Data Governance. Interview Guide. **Supplementary Material 2.** Survey on sensitive data and international health data transfers. Stakeholder Engagement for Trustworthy Data Governance. **Supplementary Material 3.** Stakeholder Engagement for Trustworthy Data Governance: Defining Sensitive Data and Developing Guidance for the Use and Transfer of Potentially Sensitive Health Data. Workshop Schedule. **Supplementary Material 4.** Table: Values, value statements and descriptions from the SHAPES framework that were voted by the stakeholders as applicable in the collection, transfer and use of data in DHT.

## Data Availability

All data generated or analysed during this study are included in this published article and its supplementary information files.
