# Supplementary material for: An ethical code for collecting, using and transferring sensitive health data: outcomes of a modified Policy Delphi process in Singapore
Source: BMC Med Ethics. 2023 Oct 4;24:78. doi: 10.1186/s12910-023-00952-7 (PMC10552227; doi:10.1186/s12910-023-00952-7)
Supplement: Supplementary file 1 — Additional file 1: Supplementary Material 1. Stakeholder Engagement for Trustworthy Data Governance. Interview Guide. Supplementary Material 2. Survey on sensitive data and international health data transfers. Stakeholder Engagement for Trustworthy Data Governance. Supplementary Material 3. Stakeholder Engagement for Trustworthy Data Governance: Defining Sensitive Data and Developing Guidance for the Use and Transfer of Potentially Sensitive Health Data. Workshop Schedule. Supplementary Material 4. Table: Values, value statements and descriptions from the SHAPES framework that were voted by the stakeholders as applicable in the collection, transfer and use of data in DHT. [file 12910_2023_952_MOESM1_ESM.docx]

**Supplementary Material 1**

## Stakeholder Engagement for Trustworthy Data Governance

## Interview Guide

1. **Introduction and build rapport**

- Discuss participants professional background and experience with data research, if any.

1. **Health data sensitivity**

- Explore what types of health-related information participants would consider as ‘sensitive’ (e.g. medical records, clinical diagnoses, hospital stays, mental health status, pathology test results, whole genome sequences) and contrast with potentially sensitive non-health information (e.g. financial/banking statements) and health data collected from mobile phone apps, wearable devices, IoTs, contact tracing etc.
- Probe on what makes certain types of health information ‘sensitive’ or not, if there are different levels of sensitivity, and if there should be greater levels of restrictions on who can access such information. If so, what sorts of restrictions?

1. **International data sharing for research**

- Explore participants views about sensitive and non-sensitive health information being stored on cloud servers that are accessible to international collaborators at publicly funded research institutions/universities overseas and contrast with industry collaborators having access to the data or if the data is sold to commercial company that is developing medical products
- Probe on whether the purpose of the research matters, or the country or region where the collaborators are located (e.g. the USA or EU country vs China or North Korea), the prestige or visibility of the university/institution or company
- Explore the conditions that participants would allow sensitive health information to be stored in Singapore and shared with international collaborators (e.g. IRB review, public benefit, removing personal identifiers, informed consent) – refer them to the reading material by NUS Medicine newsletter

1. **Regulation and governance**

- Establish how much participants know about how data research is regulated in Singapore and whether they believe the regulations are sufficient for sensitive health information, however that is understood.
  - The PDPA (administered by the PDPC) covers personally identifiable information (we can give examples like name, NRIC, address etc) but not if personal identifiers are removed/coded
  - The HBRA regulates all data research, requiring IRB approval. IRBs may grant exceptions if data is anonymous with no identifying information
- Probe on matters of consent and understanding about the trade-offs in requiring informed consent every time data is shared/accessed with the added costs of recontacting participants multiple times and risks of reidentification.
- Identify any non-legal measures that could be put in place to allow sharing of health information for research (both locally and overseas) without needing to reconsent each time
- Probe for the contours of what types of research is acceptable (e.g. disease specific, product development) or unacceptable (e.g. heritable genome editing, human cloning), and the sorts of public benefits that can be expected (or not) from research with patient health information.
- Probe on matters of transparency and accountability, and what measures are reasonable and feasible for researchers to access patient health information without obtaining informed consent each time

**Supplementary Material 2**

## **Survey on sensitive data and international health data transfers**

## **Stakeholder Engagement for Trustworthy Data Governance**

**INTRODUCTION**

You are invited to complete this survey as a panellist in our study “Stakeholder Engagement for Trustworthy Data Governance” study. The aim of this study is to develop a definition of sensitive health data that is applicable to researchers in Singapore and generate guidance for the ethical use and transfer of potentially sensitive health data.

This survey is the second stage of our study and follows the interviews we conducted between April-June 2022. It is divided into two parts:

Part 1: Defining sensitive data

Part 2: Guidance for the ethical use and transfer of potentially sensitive health data

Responses to this survey will be shared with panellists at the upcoming stakeholder workshop on October 3, 2022.

**TERMINOLOGY**

A few terms are used in this survey. We define them as follows:

- **Data contributor:** an individual whose data is collected, used and transferred for research. An example is a research participant in a study.
- **Health data**: Any information, which relates to the physical or mental health of an individual, or to the provision of health services to the individual.
- **Panellist**: This term refers to you, the panellist on our study.
- **Stakeholder**: an individual or entity with an interest in or is involved in research using health data. Examples include academic researchers, patient advocates, professional data users such as for-profit companies or clinicians, data regulators, data access controllers.
- **Personal identifiers**: Any piece of data about an individual who can be identified by that data.
- **De-identified data:** Data from which all personal identifiers have been removed, by stripping out any information that would allow people to determine an individual’s identity.

[next page]

Part 1: Defining sensitive data

Our preliminary analysis of the interviews suggest that what constitutes sensitive data is highly contextual and dependent on various factors that are difficult to codify. Nevertheless, Singapore’s Ministry of Health (MOH) has classified some data as sensitive irrespective of context. Examples are history of sexually transmitted diseases, mental health status, and reproductive health interventions (e.g., pregnancy termination, fertility treatments etc). Data in this framework are typically stored in hospital electronic medical records (EMR) and breaches are notifiable under the personal data protection regulations. The framework is available [here](https://www.moh.gov.sg/resources-statistics/dbn-list-2021).

In addition to this framework, our interviews suggest other types of health data may also be considered as sensitive. For example, several panellists mentioned genome sequencing data and genetic test results. These data are being collected in the [SG100k Project](https://www.npm.sg/collaborate/partners/sg100k/) and may be linked to EMRs in the [National Precision Medicine Strategy](https://www.npm.sg/). Thus, we are exploring the need for a more comprehensive list to discuss at the October workshop.

In this part of the survey, you will be asked to rate the sensitivity of different types of data and sources of information we have compiled from the MOH data classification framework and our interviews. You will also have an opportunity to add other types of potentially sensitive health information to this list. You will then be asked if data sensitivity is reduced when personal identifiers are removed in de-identification processes. We are asking this question because our interviews suggest there may be some agreement that data is sensitive when it is linked to a personally identifiable individual but less so once it is de-identified.

In the last question, we will ask you to agree/disagree with statements generated from the interviews about data sensitivity and give you another opportunity to add others you feel may be worthwhile discussing at the October workshop.

[next page]

1. Please rate how sensitive the following data are.

| **Data type** | **Highly**  **sensitive** | **Somewhat sensitive** | **Not very sensitive** | **Not sensitive at all** | **Unsure how sensitive** |
| --- | --- | --- | --- | --- | --- |
| 1. History of sexually transmitted diseases |  |  |  |  |  |
| 1. HIV infection status |  |  |  |  |  |
| 1. History of mental health disorders (e.g. schizophrenia, anxiety, depression) |  |  |  |  |  |
| 1. History of substance abuse and addiction (including drug addiction and alcoholism) |  |  |  |  |  |
| 1. History of fertility treatment or donation or gametes (i.e. eggs and sperm) |  |  |  |  |  |
| 1. History of contraceptive procedures (e.g. hysterectomy and vasectomy) |  |  |  |  |  |
| 1. History of pregnancy termination |  |  |  |  |  |
| 1. Details about the identity of organ donation recipients, transplant procedures |  |  |  |  |  |
| 1. History of domestic or sexual violence (victim) |  |  |  |  |  |
| 1. History of child abuse (victim) |  |  |  |  |  |
| 1. History of criminal offenses |  |  |  |  |  |
| 1. History of suicide or attempted suicide |  |  |  |  |  |
| 1. History of cancer |  |  |  |  |  |
| 1. Genome sequencing data |  |  |  |  |  |
| 1. Genetic test results |  |  |  |  |  |
| 1. Geo-location data on a handphone (i.e., GPS data) |  |  |  |  |  |
| 1. Medical history of common diseases (e.g. diabetes, stroke, heart attack etc) |  |  |  |  |  |
| 1. Medical images or scans of body parts |  |  |  |  |  |
| 1. Sexual orientation |  |  |  |  |  |
| 1. Gender identity |  |  |  |  |  |
| 1. Marital status |  |  |  |  |  |
| 1. Ethnicity or race |  |  |  |  |  |
| 1. Self-reported mental health status collected from phone apps |  |  |  |  |  |
| 1. A person’s direction finding ability collected from phone apps |  |  |  |  |  |
| 1. Voice recording or speech data collected from phone apps |  |  |  |  |  |
| 1. Electronic bank statements that show an individual’s account details |  |  |  |  |  |
| 1. Electronic pay slips that show an individual’s income |  |  |  |  |  |

Please state any other highly sensitive health data (missing from the list above) that you would like to add for discussion at the October workshop.

|  |
| --- |

[next page]

1. You rated the following list of data as highly sensitive. Indicate if the sensitivity of the data changes when personally identifying information is removed or de-identified. Please rate: (1) data remains highly sensitive, (2) data are somewhat less sensitive, or (3) data are no longer sensitive.

Do you have any comment(s) on the question and the list above? If so, please describe:

|  |
| --- |

[next page]

1. Please indicate how much you agree or disagree with the following statements about data sensitivity:

| **Statement** | **Strongly agree** | **Agree** | **Disagree** | **Strongly disagree** | **Unsure** |
| --- | --- | --- | --- | --- | --- |
| Data becomes more sensitive when it is linked to personal identifiers. |  |  |  |  |  |
| De-identified data on disease or health status becomes more sensitive when the disease is more severe. |  |  |  |  |  |
| De-identified data becomes more sensitive when it is linked to multiple datasets. |  |  |  |  |  |
| The sensitivity of data is subjective and differs from one person to the next. |  |  |  |  |  |
| The sensitivity of data cannot be classified precisely. |  |  |  |  |  |
| Sensitive data are information that people do not want others to know about without their consent. |  |  |  |  |  |
| Sensitive data are information that people want to control over who has access to it. |  |  |  |  |  |
| The rarer the disease, the more sensitive the data becomes. |  |  |  |  |  |
| Medical conditions associated with social stigma are sensitive. |  |  |  |  |  |
| Political climate affects data sensitivity. |  |  |  |  |  |
| Data sensitivity increases with the risk of harm to individuals in the event of a data breach. |  |  |  |  |  |
| Data sensitivity changes over time with changing societal values. |  |  |  |  |  |
| Data sensitivity increases with poor data management and protection practices. |  |  |  |  |  |
| Data sensitivity increases when used outside the scope of the intended purpose. |  |  |  |  |  |
| Data sensitivity relates to the trustworthiness of the organisations securing the data. |  |  |  |  |  |
| Data that may discriminate against employment options are sensitive. |  |  |  |  |  |
| Data that may discriminate against a person’s access to insurance are sensitive. |  |  |  |  |  |
| Data that may negatively impact the reputation of the individual are sensitive. |  |  |  |  |  |

Do you have any comment(s) on the statements above? If so, please describe:

|  |
| --- |

[next page]

Part 2: Guidance for the ethical use and transfer of potentially sensitive health data

Our preliminary analysis of the interviews is consistent with our previous studies on public attitudes toward health data sharing in Singapore. These studies have found broad conditional support for health-related data being shared with universities and healthcare institutions in Singapore for the purposes of research. However, there is less support for data being transferred out of Singapore to research institutions overseas with concerns around data security and the strength of data protection laws in the recipient country. There are also concerns around the reputation and track record of the receiving institution and the benefits for Singapore in transferring data overseas. There is an even greater reluctance for data being transferred to international commercial collaborators, even when the data are de-identified. Participants in our previous studies and current panellists have expressed concerns around commercial operators profiteering or misusing the data without any public benefits for Singapore. There are also concerns around accountability and oversight once data are transferred overseas out of Singapore’s jurisdictional authority.

Having said that, our studies suggest potentially sensitive health data may be transferred to collaborators overseas in both public and private research sectors provided certain conditions are met. They include, but not limited to data being de-identified with the highest security protocols in place, data contributors have opted in to the research and are able to withdraw consent, there are good governance systems in place that enable transparency and accountability in the use and transfer of the data, and the research is for the public good of Singapore. For more information, you may refer to our published findings:

Ballantyne, A., Lysaght, T., et al. (2022). Sharing precision medicine data with private industry: Outcomes of a citizens’ jury in Singapore. Big Data & Society. doi:10.1177/20539517221108988
([Link](https://journals.sagepub.com/doi/full/10.1177/20539517221108988))

Lysaght, T., Ballantyne, A., et al. (2021). Trust and Trade-Offs in Sharing Data for Precision Medicine: A National Survey of Singapore. Journal of Personalized Medicine, 11(9), 921. doi: 10.3390/jpm11090921
([Link](https://www.mdpi.com/2075-4426/11/9/921))

Lysaght, T., Ballantyne, A., et al. (2020). “Who is Watching the Watchdog?”: Ethical Perspectives of Sharing Health-related Data for Precision Medicine in Singapore. doi: 10.1186/s12910-020-00561-8
([Link](https://bmcmedethics.biomedcentral.com/articles/10.1186/s12910-020-00561-8))

[next page]

For Part 2 of this survey, we have generated lists of statements from the interviews that could guide researchers working with potentially sensitive health data in Singapore. These statements are organised under 16 values identified in the SHAPES Big Data Ethics Framework (see [here](https://link.springer.com/article/10.1007/s41649-019-00099-x)). These 16 ethical values are:

1. Accountability
2. Autonomy
3. Consistency
4. Engagement
5. Harm Minimization
6. Integrity
7. Justice
8. Privacy
9. Proportionality
10. Public Benefit
11. Reasonableness
12. Reflexivity
13. Solidarity
14. Stewardship
15. Transparency 
16. Trustworthiness

We would like feedback from you on each statement with respect to its 1) desirability, and 2) feasibility for an ethical code to guide the use and transfer of potentially sensitive health data. You should rate each statement as what you think should be the ethical standard for guiding the use and transfer of potentially sensitive health data, and not based on current data use and transfer practices. In the following section, we will provide a definition for each value and ask you to rate how much you agree or disagree with 2-4 statements as desirable and feasible expressions of that value.

• Desirable: The statement is a good way to express this value.
• Feasible: The statement is practical to implement.

At the bottom of each page, you will have the opportunity to comment on the statements and to suggest alternative ones. Statements that are rated highly desirable and highly feasible will be discussed for further refinement at the October workshop.

[next page]

**Value 1 - Accountability**

**Definition:** Accountability refers to the ability to scrutinise judgements, decisions and actions, and for decision-makers to be held responsible for their consequences.

***Definitions of "desirable" and "feasible"***
Desirable: The statement is a good way to express this value.
Feasible: The statement is practical to implement.

**Statement 1: We are responsible for the data in our collection and any breaches to our data security protocols.**

| **Statement** | **Strongly agree** | **Agree** | **Disagree** | **Strongly disagree** | **Don’t know** |
| --- | --- | --- | --- | --- | --- |
| This statement is DESIRABLE |  |  |  |  |  |
| This statement is FEASIBLE. |  |  |  |  |  |

**Statement 2: We are responsible for any data in our collection.**

| **Statement** | **Strongly agree** | **Agree** | **Disagree** | **Strongly disagree** | **Don’t know** |
| --- | --- | --- | --- | --- | --- |
| This statement is DESIRABLE |  |  |  |  |  |
| This statement is FEASIBLE. |  |  |  |  |  |

**Statement 3: We ensure our research partners are accountable for the use and transfer of data from our collection through enforceable contractual agreements.**

| **Statement** | **Strongly agree** | **Agree** | **Disagree** | **Strongly disagree** | **Don’t know** |
| --- | --- | --- | --- | --- | --- |
| This statement is DESIRABLE |  |  |  |  |  |
| This statement is FEASIBLE. |  |  |  |  |  |

**Statement 4: We ensure our research partners are appropriately accredited before we transfer data from our collection.**

| **Statement** | **Strongly agree** | **Agree** | **Disagree** | **Strongly disagree** | **Don’t know** |
| --- | --- | --- | --- | --- | --- |
| This statement is DESIRABLE |  |  |  |  |  |
| This statement is FEASIBLE. |  |  |  |  |  |

Is there anything you would add to these statements? If so, please describe:

|  |
| --- |

[next page]

**Value 2 - Autonomy**

**Definition:** Autonomy is defined as the capacity of a person or group to be self-determining.

***Definitions of "desirable" and "feasible"***
Desirable: The statement is a good way to express this value.
Feasible: The statement is practical to implement.

**Statement 5: We collect, use and transfer data for research with the informed consent from contributors.**

| **Statement** | **Strongly agree** | **Agree** | **Disagree** | **Strongly disagree** | **Don’t know** |
| --- | --- | --- | --- | --- | --- |
| This statement is DESIRABLE |  |  |  |  |  |
| This statement is FEASIBLE. |  |  |  |  |  |

**Statement 6: We respect the right of contributors to withdraw consent to use and transfer data in our collection.**

| **Statement** | **Strongly agree** | **Agree** | **Disagree** | **Strongly disagree** | **Don’t know** |
| --- | --- | --- | --- | --- | --- |
| This statement is DESIRABLE |  |  |  |  |  |
| This statement is FEASIBLE. |  |  |  |  |  |

**Statement 7: We obtain consent from contributors when there are changes in how data in our collection is being used and transferred for research.**

| **Statement** | **Strongly agree** | **Agree** | **Disagree** | **Strongly disagree** | **Don’t know** |
| --- | --- | --- | --- | --- | --- |
| This statement is DESIRABLE |  |  |  |  |  |
| This statement is FEASIBLE. |  |  |  |  |  |

**Statement 8: We provide clear and comprehensible information to prospective data contributors before we collect, use or transfer data.**

| **Statement** | **Strongly agree** | **Agree** | **Disagree** | **Strongly disagree** | **Don’t know** |
| --- | --- | --- | --- | --- | --- |
| This statement is DESIRABLE |  |  |  |  |  |
| This statement is FEASIBLE. |  |  |  |  |  |

Is there anything you would add to these statements? If so, please describe:

|  |
| --- |

[next page]

**Value 3 - Consistency**

**Definition:** In the absence of relevant differences between two or more situations, consistency requires that the same standards be applied across them. While consistency in decision-making may be regarded as valuable in its own right, adherence to a practice of consistency may help actors to secure other values, such as fairness and trustworthiness.

***Definitions of "desirable" and "feasible"***
Desirable: The statement is a good way to express this value.
Feasible: The statement is practical to implement.

**Statement 9: We take the same care when transferring data to research partners in Singapore and overseas.**

| **Statement** | **Strongly agree** | **Agree** | **Disagree** | **Strongly disagree** | **Don’t know** |
| --- | --- | --- | --- | --- | --- |
| This statement is DESIRABLE |  |  |  |  |  |
| This statement is FEASIBLE. |  |  |  |  |  |

**Statement 10: We pay due care and attention to all potentially sensitive data.**

| **Statement** | **Strongly agree** | **Agree** | **Disagree** | **Strongly disagree** | **Don’t know** |
| --- | --- | --- | --- | --- | --- |
| This statement is DESIRABLE |  |  |  |  |  |
| This statement is FEASIBLE. |  |  |  |  |  |

**Statement 11: We protect all data in our collection.**

| **Statement** | **Strongly agree** | **Agree** | **Disagree** | **Strongly disagree** | **Don’t know** |
| --- | --- | --- | --- | --- | --- |
| This statement is DESIRABLE |  |  |  |  |  |
| This statement is FEASIBLE. |  |  |  |  |  |

Is there anything you would add to these statements? If so, please describe:

|  |
| --- |

[next page]

**Value 4 - Engagement**

**Definition:** Engagement is the meaningful involvement of stakeholders in the design and conduct of the data activities. Engagement goes beyond the dissemination of information and requires that data activities have been influenced in some way by the views of stakeholders.

***Definitions of "desirable" and "feasible"***
Desirable: The statement is a good way to express this value.
Feasible: The statement is practical to implement.

**Statement 12: We continually involve data contributors and communicate with them about our research activities.**

| **Statement** | **Strongly agree** | **Agree** | **Disagree** | **Strongly disagree** | **Don’t know** |
| --- | --- | --- | --- | --- | --- |
| This statement is DESIRABLE |  |  |  |  |  |
| This statement is FEASIBLE. |  |  |  |  |  |

**Statement 13: We seek out input from stakeholders on how we use and transfer data in our collection.**

| **Statement** | **Strongly agree** | **Agree** | **Disagree** | **Strongly disagree** | **Don’t know** |
| --- | --- | --- | --- | --- | --- |
| This statement is DESIRABLE |  |  |  |  |  |
| This statement is FEASIBLE. |  |  |  |  |  |

**Statement 14: We involve data contributors in the design and conduct of our research.**

| **Statement** | **Strongly agree** | **Agree** | **Disagree** | **Strongly disagree** | **Don’t know** |
| --- | --- | --- | --- | --- | --- |
| This statement is DESIRABLE |  |  |  |  |  |
| This statement is FEASIBLE. |  |  |  |  |  |

Is there anything you would add to these statements? If so, please describe:

|  |
| --- |

[next page]

**Value 5 - Harm Minimisation**

**Definition:** Harm minimisation involves reducing the possibility of real or perceived harms (physical, economic, psychological, emotional, or reputational) to persons.

***Definitions of "desirable" and "feasible"***
Desirable: The statement is a good way to express this value.
Feasible: The statement is practical to implement.

**Statement 15: We reduce the risks of re-identifying contributors when using or transferring data in our collection for research.**

| **Statement** | **Strongly agree** | **Agree** | **Disagree** | **Strongly disagree** | **Don’t know** |
| --- | --- | --- | --- | --- | --- |
| This statement is DESIRABLE |  |  |  |  |  |
| This statement is FEASIBLE. |  |  |  |  |  |

**Statement 16: We safeguard the identities of contributors in our data collection.**

| **Statement** | **Strongly agree** | **Agree** | **Disagree** | **Strongly disagree** | **Don’t know** |
| --- | --- | --- | --- | --- | --- |
| This statement is DESIRABLE |  |  |  |  |  |
| This statement is FEASIBLE. |  |  |  |  |  |

**Statement 17: We comply with the best standards applicable to data collection, use and transfer to reduce potential harms to groups of individuals.**

| **Statement** | **Strongly agree** | **Agree** | **Disagree** | **Strongly disagree** | **Don’t know** |
| --- | --- | --- | --- | --- | --- |
| This statement is DESIRABLE |  |  |  |  |  |
| This statement is FEASIBLE. |  |  |  |  |  |

Is there anything you would add to these statements? If so, please describe:

|  |
| --- |

[next page]

**Value 6 - Integrity**

**Definition:** Integrity refers to a feature or property of those acting in accordance with personal and/or accepted scientific and professional values and commitments.

***Definitions of "desirable" and "feasible"***
Desirable: The statement is a good way to express this value.
Feasible: The statement is practical to implement.

**Statement 18: We ensure the use and transfer of data for research is consistent with what contributors have consented to.**

| **Statement** | **Strongly agree** | **Agree** | **Disagree** | **Strongly disagree** | **Don’t know** |
| --- | --- | --- | --- | --- | --- |
| This statement is DESIRABLE |  |  |  |  |  |
| This statement is FEASIBLE. |  |  |  |  |  |

**Statement 19: We will not sell the data in our collection to third parties.**

| **Statement** | **Strongly agree** | **Agree** | **Disagree** | **Strongly disagree** | **Don’t know** |
| --- | --- | --- | --- | --- | --- |
| This statement is DESIRABLE |  |  |  |  |  |
| This statement is FEASIBLE. |  |  |  |  |  |

**Statement 20: Our data collection, use and transfer meet the highest standard for scientific research.**

| **Statement** | **Strongly agree** | **Agree** | **Disagree** | **Strongly disagree** | **Don’t know** |
| --- | --- | --- | --- | --- | --- |
| This statement is DESIRABLE |  |  |  |  |  |
| This statement is FEASIBLE. |  |  |  |  |  |

Is there anything you would add to these statements? If so, please describe:

|  |
| --- |

[next page]

**Value 7 - Justice**

**Definition:** Justice consists in treating individuals and groups fairly and with respect. This includes the fair distribution of benefits and burdens of data activities (collection, storage, use, linkage, and sharing) and attention to issues of equity.

***Definitions of "desirable" and "feasible"***
Desirable: The statement is a good way to express this value.
Feasible: The statement is practical to implement.

**Statement 21: We include underserved communities and minority groups in our data collections.**

| **Statement** | **Strongly agree** | **Agree** | **Disagree** | **Strongly disagree** | **Don’t know** |
| --- | --- | --- | --- | --- | --- |
| This statement is DESIRABLE |  |  |  |  |  |
| This statement is FEASIBLE. |  |  |  |  |  |

**Statement 22: We amend for any harms caused to contributors who are identified from the use or transfer of data in our collection.**

| **Statement** | **Strongly agree** | **Agree** | **Disagree** | **Strongly disagree** | **Don’t know** |
| --- | --- | --- | --- | --- | --- |
| This statement is DESIRABLE |  |  |  |  |  |
| This statement is FEASIBLE. |  |  |  |  |  |

**Statement 23: We ensure the use and transfer of data in our collection does not unduly burden our data contributors.**

| **Statement** | **Strongly agree** | **Agree** | **Disagree** | **Strongly disagree** | **Don’t know** |
| --- | --- | --- | --- | --- | --- |
| This statement is DESIRABLE |  |  |  |  |  |
| This statement is FEASIBLE. |  |  |  |  |  |

Is there anything you would add to these statements? If so, please describe:

|  |
| --- |

[next page]

**Value 8 - Privacy**

**Definition:** For the purposes of this Framework, privacy refers to controlling access to information about persons. Privacy is valuable because the ability to control access to information about persons promotes certain core interests that we have as individuals and groups. These are wide-ranging but include identity interests and the promotion of human autonomous decision-making, as well as freedom from potential harms such as discrimination and stigmatisation that may arise from our data being disclosed. This control may be exercised directly by individuals to whom the data pertains, or by designated persons, such as data custodians whose decisions aim to promote those core individual and group interests.

***Definitions of "desirable" and "feasible"***
Desirable: The statement is a good way to express this value.
Feasible: The statement is practical to implement.

**Statement 24: We only use and transfer de-identified data in our collection for research.**

| **Statement** | **Strongly agree** | **Agree** | **Disagree** | **Strongly disagree** | **Don’t know** |
| --- | --- | --- | --- | --- | --- |
| This statement is DESIRABLE |  |  |  |  |  |
| This statement is FEASIBLE. |  |  |  |  |  |

**Statement 25: We respect the privacy of data contributors.**

| **Statement** | **Strongly agree** | **Agree** | **Disagree** | **Strongly disagree** | **Don’t know** |
| --- | --- | --- | --- | --- | --- |
| This statement is DESIRABLE |  |  |  |  |  |
| This statement is FEASIBLE. |  |  |  |  |  |

**Statement 26: We do not store or transfer identifiable information of data contributors.**

| **Statement** | **Strongly agree** | **Agree** | **Disagree** | **Strongly disagree** | **Don’t know** |
| --- | --- | --- | --- | --- | --- |
| This statement is DESIRABLE |  |  |  |  |  |
| This statement is FEASIBLE. |  |  |  |  |  |

**Statement 27: We transfer de-identified data securely.**

| **Statement** | **Strongly agree** | **Agree** | **Disagree** | **Strongly disagree** | **Don’t know** |
| --- | --- | --- | --- | --- | --- |
| This statement is DESIRABLE |  |  |  |  |  |
| This statement is FEASIBLE. |  |  |  |  |  |

Is there anything you would add to these statements? If so, please describe:

|  |
| --- |

[next page]

**Value 9 - Proportionality**

**Definition:** Proportionality is a consideration in decision-making that requires that the means are necessary and appropriate in relation to the end that is being pursued and being cognisant of the competing interests at hand.

***Definitions of "desirable" and "feasible"***
Desirable: The statement is a good way to express this value.
Feasible: The statement is practical to implement.

**Statement 28: We take greater care with data that is potentially sensitive.**

| **Statement** | **Strongly agree** | **Agree** | **Disagree** | **Strongly disagree** | **Don’t know** |
| --- | --- | --- | --- | --- | --- |
| This statement is DESIRABLE |  |  |  |  |  |
| This statement is FEASIBLE. |  |  |  |  |  |

**Statement 29: The more sensitive the data we collect, the greater security protocols we put in place.**

| **Statement** | **Strongly agree** | **Agree** | **Disagree** | **Strongly disagree** | **Don’t know** |
| --- | --- | --- | --- | --- | --- |
| This statement is DESIRABLE |  |  |  |  |  |
| This statement is FEASIBLE. |  |  |  |  |  |

**Statement 30: We only collect, use and transfer data that are necessary and relevant to the intended research purpose.**

| **Statement** | **Strongly agree** | **Agree** | **Disagree** | **Strongly disagree** | **Don’t know** |
| --- | --- | --- | --- | --- | --- |
| This statement is DESIRABLE |  |  |  |  |  |
| This statement is FEASIBLE. |  |  |  |  |  |

Is there anything you would add to these statements? If so, please describe:

|  |
| --- |

[next page]

**Value 10 - Public Benefit**

**Definition:** Public benefit is the overall good that society as a whole receives from a given project. This includes consideration of effects on wellbeing, distribution, societal cohesion, human rights, and other sources of value to society. It may not be possible to measure these factors by the same standards, so some judgement and critical analysis will be required in determining what is publicly beneficial.

***Definitions of "desirable" and "feasible"***
Desirable: The statement is a good way to express this value.
Feasible: The statement is practical to implement.

**Statement 31: Our health data research is intended to benefit patients in Singapore.**

| **Statement** | **Strongly agree** | **Agree** | **Disagree** | **Strongly disagree** | **Don’t know** |
| --- | --- | --- | --- | --- | --- |
| This statement is DESIRABLE |  |  |  |  |  |
| This statement is FEASIBLE. |  |  |  |  |  |

**Statement 32: We use and transfer data with the intention of benefiting future patients.**

| **Statement** | **Strongly agree** | **Agree** | **Disagree** | **Strongly disagree** | **Don’t know** |
| --- | --- | --- | --- | --- | --- |
| This statement is DESIRABLE |  |  |  |  |  |
| This statement is FEASIBLE. |  |  |  |  |  |

**Statement 33: We only transfer data for research that can benefit Singapore.**

| **Statement** | **Strongly agree** | **Agree** | **Disagree** | **Strongly disagree** | **Don’t know** |
| --- | --- | --- | --- | --- | --- |
| This statement is DESIRABLE |  |  |  |  |  |
| This statement is FEASIBLE. |  |  |  |  |  |

Is there anything you would add to these statements? If so, please describe:

|  |
| --- |

[next page]

**Value 11 - Reasonableness**

**Definition:** Reasonableness means appealing to reasons and values that are widely recognised as relevant and fair.

***Definitions of "desirable" and "feasible"***
Desirable: The statement is a good way to express this value.
Feasible: The statement is practical to implement.

**Statement 34: We use good judgement in how we use and transfer data for research.**

| **Statement** | **Strongly agree** | **Agree** | **Disagree** | **Strongly disagree** | **Don’t know** |
| --- | --- | --- | --- | --- | --- |
| This statement is DESIRABLE |  |  |  |  |  |
| This statement is FEASIBLE. |  |  |  |  |  |

**Statement 35: We use and transfer data in our collections with good reasons.**

| **Statement** | **Strongly agree** | **Agree** | **Disagree** | **Strongly disagree** | **Don’t know** |
| --- | --- | --- | --- | --- | --- |
| This statement is DESIRABLE |  |  |  |  |  |
| This statement is FEASIBLE. |  |  |  |  |  |

**Statement 36: We only transfer data overseas when we need to leverage on foreign capabilities to build local capabilities.**

| **Statement** | **Strongly agree** | **Agree** | **Disagree** | **Strongly disagree** | **Don’t know** |
| --- | --- | --- | --- | --- | --- |
| This statement is DESIRABLE |  |  |  |  |  |
| This statement is FEASIBLE. |  |  |  |  |  |

**Statement 37: When determining data use/access, our justifications appeal only to reasons our stakeholders could generally accept no matter their backgrounds.**

| **Statement** | **Strongly agree** | **Agree** | **Disagree** | **Strongly disagree** | **Don’t know** |
| --- | --- | --- | --- | --- | --- |
| This statement is DESIRABLE |  |  |  |  |  |
| This statement is FEASIBLE. |  |  |  |  |  |

Is there anything you would add to these statements? If so, please describe:

|  |
| --- |

[next page]

**Value 12 – Reflexivity**

**Definition:** Reflexivity refers to the process of reflecting on and responding to the limitations and uncertainties embedded in knowledge, information, evidence, and data. This includes being alert to competing and conflicting personal, professional, and organisational interests and to the management of associated biases. Reflexive institutions revise or create new policies and systems that change institutional processes and prompt further reflection and response.

***Definitions of "desirable" and "feasible"***
Desirable: The statement is a good way to express this value.
Feasible: The statement is practical to implement.

**Statement 38: We look for new ways and resources to minimize risks to our data contributors.**

| **Statement** | **Strongly agree** | **Agree** | **Disagree** | **Strongly disagree** | **Don’t know** |
| --- | --- | --- | --- | --- | --- |
| This statement is DESIRABLE |  |  |  |  |  |
| This statement is FEASIBLE. |  |  |  |  |  |

**Statement 39: We constantly monitor our security protocols.**

| **Statement** | **Strongly agree** | **Agree** | **Disagree** | **Strongly disagree** | **Don’t know** |
| --- | --- | --- | --- | --- | --- |
| This statement is DESIRABLE |  |  |  |  |  |
| This statement is FEASIBLE. |  |  |  |  |  |

**Statement 40: We acknowledge there are risks of harms to data contributors that are unknown and/or out of our control.**

| **Statement** | **Strongly agree** | **Agree** | **Disagree** | **Strongly disagree** | **Don’t know** |
| --- | --- | --- | --- | --- | --- |
| This statement is DESIRABLE |  |  |  |  |  |
| This statement is FEASIBLE. |  |  |  |  |  |

Is there anything you would add to these statements? If so, please describe:

|  |
| --- |

[next page]

**Value 13 - Solidarity**

**Definition:** Solidarity is the commitment among persons with recognised morally relevant sameness or similarity to sharing costs and benefits for the good of a group, community, nation, or global population.

***Definitions of "desirable" and "feasible"***
Desirable: The statement is a good way to express this value.
Feasible: The statement is practical to implement.

**Statement 41: The data we collect, use and transfer are sourced from the populations our research is intended to benefit.**

| **Statement** | **Strongly agree** | **Agree** | **Disagree** | **Strongly disagree** | **Don’t know** |
| --- | --- | --- | --- | --- | --- |
| This statement is DESIRABLE |  |  |  |  |  |
| This statement is FEASIBLE. |  |  |  |  |  |

**Statement 42: We recognise the risks to our contributors when we use and transfer the data in our collection.**

| **Statement** | **Strongly agree** | **Agree** | **Disagree** | **Strongly disagree** | **Don’t know** |
| --- | --- | --- | --- | --- | --- |
| This statement is DESIRABLE |  |  |  |  |  |
| This statement is FEASIBLE. |  |  |  |  |  |

**Statement 43: We will not use or transfer data to regions that have fewer legal protections primarily for ease of doing research there.**

| **Statement** | **Strongly agree** | **Agree** | **Disagree** | **Strongly disagree** | **Don’t know** |
| --- | --- | --- | --- | --- | --- |
| This statement is DESIRABLE |  |  |  |  |  |
| This statement is FEASIBLE. |  |  |  |  |  |

Is there anything you would add to these statements? If so, please describe:

|  |
| --- |

[next page]

**Value 14 - Stewardship**

**Definition:** Stewardship reflects a relationship with things, such as data, to promote twin objectives of taking care of the object of attention as well as seeking actively to promote its value and utility. It involves guiding others with prudence and care across one or more endeavours—without which there is risk of impairment or harm—and with a view to collective betterment.

***Definitions of "desirable" and "feasible"***
Desirable: The statement is a good way to express this value.
Feasible: The statement is practical to implement.

**Statement 44: We collect, use and transfer data with the greatest care for the intended research purpose.**

| **Statement** | **Strongly agree** | **Agree** | **Disagree** | **Strongly disagree** | **Don’t know** |
| --- | --- | --- | --- | --- | --- |
| This statement is DESIRABLE |  |  |  |  |  |
| This statement is FEASIBLE. |  |  |  |  |  |

**Statement 45: We recognise the value of health data as a public good and the need to continuously safeguard the data in our collection.**

| **Statement** | **Strongly agree** | **Agree** | **Disagree** | **Strongly disagree** | **Don’t know** |
| --- | --- | --- | --- | --- | --- |
| This statement is DESIRABLE |  |  |  |  |  |
| This statement is FEASIBLE. |  |  |  |  |  |

**Statement 46: We ensure data we collect are only used or transferred for the research purposes that fall within the consented scope.**

| **Statement** | **Strongly agree** | **Agree** | **Disagree** | **Strongly disagree** | **Don’t know** |
| --- | --- | --- | --- | --- | --- |
| This statement is DESIRABLE |  |  |  |  |  |
| This statement is FEASIBLE. |  |  |  |  |  |

Is there anything you would add to these statements? If so, please describe:

|  |
| --- |

[next page]

**Value 15 - Transparency**

**Definition:** Transparency is openness to public scrutiny of decision-making, processes, and actions. Transparency helps to demonstrate respect for persons and contributes to trustworthiness.

***Definitions of "desirable" and "feasible"***
Desirable: The statement is a good way to express this value.
Feasible: The statement is practical to implement.

**Statement 47: We are transparent about how we collect, use and transfer data for research.**

| **Statement** | **Strongly agree** | **Agree** | **Disagree** | **Strongly disagree** | **Don’t know** |
| --- | --- | --- | --- | --- | --- |
| This statement is DESIRABLE |  |  |  |  |  |
| This statement is FEASIBLE. |  |  |  |  |  |

**Statement 48: We promptly report breaches to our security protocols to the relevant authorities and contact data contributors about the incident.**

| **Statement** | **Strongly agree** | **Agree** | **Disagree** | **Strongly disagree** | **Don’t know** |
| --- | --- | --- | --- | --- | --- |
| This statement is DESIRABLE |  |  |  |  |  |
| This statement is FEASIBLE. |  |  |  |  |  |

**Statement 49: We share information about the outcomes of our research with data contributors.**

| **Statement** | **Strongly agree** | **Agree** | **Disagree** | **Strongly disagree** | **Don’t know** |
| --- | --- | --- | --- | --- | --- |
| This statement is DESIRABLE |  |  |  |  |  |
| This statement is FEASIBLE. |  |  |  |  |  |

Is there anything you would add to these statements? If so, please describe:

|  |
| --- |

[next page]

**Value 16 – Trustworthiness**

**Definition:** Trustworthiness is the property of being worthy of trust. It is a value that applies not only to individuals, organisations, governments, and institutions, but also to data, evidence, and systems. It can manifest procedurally as being transparent and truthful, reliable and consistent, or dependable.

***Definitions of "desirable" and "feasible"***
Desirable: The statement is a good way to express this value.
Feasible: The statement is practical to implement.

**Statement 50: We assess the trustworthiness of research partners overseas according to the data protection regulations in the country they operate in.**

| **Statement** | **Strongly agree** | **Agree** | **Disagree** | **Strongly disagree** | **Don’t know** |
| --- | --- | --- | --- | --- | --- |
| This statement is DESIRABLE |  |  |  |  |  |
| This statement is FEASIBLE. |  |  |  |  |  |

**Statement 51: We assess the trustworthiness of research partners according to their track record and reputation.**

| **Statement** | **Strongly agree** | **Agree** | **Disagree** | **Strongly disagree** | **Don’t know** |
| --- | --- | --- | --- | --- | --- |
| This statement is DESIRABLE |  |  |  |  |  |
| This statement is FEASIBLE. |  |  |  |  |  |

**Statement 52: We recognise that data contributors trust us to secure, use and transfer data in our collection appropriately.**

| **Statement** | **Strongly agree** | **Agree** | **Disagree** | **Strongly disagree** | **Don’t know** |
| --- | --- | --- | --- | --- | --- |
| This statement is DESIRABLE |  |  |  |  |  |
| This statement is FEASIBLE. |  |  |  |  |  |

**Statement 53: We demonstrate our trustworthiness by using and transferring data in our collection only for the intended purposes of research.**

| **Statement** | **Strongly agree** | **Agree** | **Disagree** | **Strongly disagree** | **Don’t know** |
| --- | --- | --- | --- | --- | --- |
| This statement is DESIRABLE |  |  |  |  |  |
| This statement is FEASIBLE. |  |  |  |  |  |

Is there anything you would add to these statements? If so, please describe:

|  |
| --- |

**Supplementary Material 3**

## Stakeholder Engagement for Trustworthy Data Governance: Defining Sensitive Data and Developing Guidance for the Use and Transfer of Potentially Sensitive Health Data

Workshop Schedule

Date : 3^rd^ October 2022 (Monday)

Time : 8:30a.m. – 5.00p.m.

Venue : Pinnacle Room (level 16), CREATE Tower NUS, 1 CREATE Way, Singapore 138602

| **Time** | **Activity** | **Presenter(s) / Facilitator(s)** |
| --- | --- | --- |
| 8.30am – 9.00am  (30 mins) | Panellists’ registration  Coffee & tea (foyer) | - |
| 9.00am – 9.10am  (10 mins) | Welcome & introduction to workshop  Housekeeping | Tamra Lysaght, National University of Singapore  Bernadette Richards, The University of Queensland |
| 9.10am – 9.40am  (20 mins) | Icebreaking activities | Andrew Lau,  Projective Insights |
| 9.40am – 11.00am  (1 hr 20 mins) | Round #01 Deliberation (Part 1):  Presentation and Q&A on survey findings  Defining sensitive data | Andrew Lau  Chan Hui Yun, National University of Singapore  Tamra Lysaght  Bernadette Richards |
| 11.00am – 11.30am  (30 mins) | Morning tea  (Foyer) | - |
| 11.30am – 12.30pm  (1 hr) | Round #01 Deliberation (Part 2):  Defining sensitive data  (Breakout rooms) | Tamra Lysaght  Bernadette Richards  Andrew Lau |
| 12.30pm – 1.15pm  (45 mins) | Lunch  (Sky Garden at level 13) | - |
| 1.15pm – 1.30pm  (15 mins) | Pre-deliberation briefing:  Overview of AFIRRM principles and big data ethics framework with Q&A | Effy Vayena, Swiss Institute of Technology (ETH)  Tamra Lysaght  Bernadette Richards |
| 1:30pm – 3.00pm  (1 hr 30 mins) | Round #02 Deliberation:  Presentation and Q&A on survey findings  Generating guidance for international health data transfer and use  (Breakout rooms) | Andrew Lau  Chan Hui Yun  Effy Vayena  Tamra Lysaght  Bernadette Richards |
| 3.00pm – 3.30pm  (30 mins) | Afternoon tea  (Foyer) | - |
| 3.30pm – 4.45pm  (1 hr 15 mins) | Round #02 Deliberation:  Generating guidance for international health data transfer and use | Andrew Lau  Tamra Lysaght  Effy Vayena  Chan Hui Yun  Bernadette Richards |
| 4.45pm – 5.00pm  (15 mins) | Final remarks and closing | Tamra Lysaght |

**Supplementary Material 4**

## Table: Values, value statements and descriptions from the SHAPES framework that were voted by the stakeholders as applicable in the collection, transfer and use of data in DHT.

| **Ethics Framework for Big Data in Health and Research** | | | |
| --- | --- | --- | --- |
| No. | Category | Value | **Value statement** and description |
| 1 | Substantive value | Autonomy | **We respect the rights of the data contributors to decide whether we collect, use or transfer their data.**  Description: We acknowledge that individuals have legal and moral rights to control access to information about them. We show respect to individuals by obtaining consent to collect, use and transfer their data for broad research purposes. Unless otherwise required by the law, we will obtain a waiver from an IRB for any secondary research that broadly falls within the scope of the contributors’ original consent. We will, to the best of our ability, respect the rights of data contributors to withdraw from research where it is practical and feasible to remove them from our databases. |
| 2 | Substantive value | Fairness and Equality | **We minimise the burdens of data collection on our contributors and strive to be inclusive and unbiased when collecting, using and transferring data.**  *Description*: We acknowledge there are costs to contributing data for research and we aim to minimise them by only collecting, using and transferring information we need to achieve our research aims. We also strive to include underserved communities and minority groups in our data collection to facilitate representativeness and promote fair distribution and access to the benefits of our research. |
| 3 | Substantive value | Harm Minimisation | **We strive to reduce harm to, and protect the privacy of, groups and individuals by complying with the best practices applicable to safeguard the identities of data contributors and responding appropriately if harm occurs.**  *Description*: We recognise that harms (e.g., financial, reputational, discriminatory, stigma etc) arising from data-intensive research, even if unlikely, could be detrimental to the livelihood and well-being of individuals, groups and populations. We strive to reduce the risks of these harms by implementing best practices and mitigation strategies to protect the identity of data contributors. When they do occur, we are committed to responding appropriately to remedy any harms caused. |
| 4 | Substantive value | Integrity | **We collect, use and transfer data in accordance with what the data contributors have consented to and the prescribed standards for research.**  *Description*: We conduct research according to widely-accepted standards and will not sell any data in our collection to third parties without the consent of data contributors. |
| 5 | Substantive value | Proportionality | **The more sensitive the data we collect, the greater the data protection and security controls we put in place.**  *Description*: We take a tiered approach to managing risks in collecting, using and transferring data in our collection. We put in place stronger IT infrastructure (i.e., technical security measures) and data protection practices to protect privacy as data sensitivity and risk of harm increases. |
| 6 | Substantive value | Public Benefit | **We use and transfer data with the intention of benefitting health systems, patients, and populations globally.**  *Description*: Our research is intended to benefit patients and populations through improved health systems and community-based care, both in Singapore and overseas. |
| 7 | Substantive value | Solidarity | **We recognise the risks to our contributors when we use and transfer the data in our collection and we will not use or transfer data to regions that have inadequate legal protection.**  *Description*: We acknowledge the risks of research to data contributors and will not exploit populations in countries with weak or inadequate data privacy laws simply for ease of doing research there. |
| 8 | Substantive value | Stewardship | **We recognise the value of health data as a public good and we collect, use and transfer data with the appropriate care for the intended research purpose.**  *Description*: We recognise health data is a public good and we have responsibilities to continually safeguard the data in our collection and ensure our research is in the public interest. |
| 9 | Procedural values | Accountability | **We are accountable and responsible for the use and transfer of data from our collection and, to the best of our ability, ensure that our partners comply with the relevant laws and regulations.**  *Description*: We take responsibility for the use and transfer of data in our collection and will be accountable for any breaches in our data security protocols. We also ensure that our partners are reputable and comply with the relevant laws and regulations for the use and transfer of data from our collection through enforceable contractual agreements. |
| 10 | Procedural values | Consistency | **We pay due care and attention to all data we collect, use and transfer.**  *Description*: We take responsibility for the data in our collection regardless of its sensitivity. We take the same care when transferring data to research partners in Singapore and overseas. |
| 11 | Procedural values | Engagement | **We seek input from stakeholders in the design and conduct of our research, and how we collect, use and transfer data in our collection.**  *Description*: We engage stakeholders in the design and conduct of our research and aspire to continually involve data contributors in this process and communicate with them about our research activities where practical. |
| 12 | Procedural values | Reflexivity | **We acknowledge there are risks of harms to data contributors that are unknown to us or out of our control and we will proactively monitor and manage those risks.**  *Description*: We acknowledge there are limits to what we know and do not know, and it is our responsibility to look for new ways and resources to minimise risks to our data contributors. |
| 13 | Procedural values | Transparency | **We submit to regulatory scrutiny of the collection, use and transfer of data and we promptly report breaches.**  *Description*: We conduct audits on how we collect, use and transfer data for research. We proactively monitor for risks and promptly report breaches to our security protocols to the relevant authorities and contact data contributors about the incident. Where feasible, we share outcomes of our research with data contributors. |
| 14 | Procedural values | Trustworthiness | **We recognise that data contributors trust us and our partners to secure use and transfer data in our collection appropriately and reasonably.**  *Description*: We demonstrate our trustworthiness by using and transferring data in our collection only for the intended purposes of research. We take reasonable measures to assess the trustworthiness of research partners according to their track record and reputation, including the data protection regulations in the country they operate. |
